# Benefit of intravitreal injections in patients with sub-threshold baseline visual acuity: a retrospective single-centre study

**DOI:** 10.1007/s00417-023-05989-3

**Published:** 2023-03-16

**Authors:** Nina-Antonia Grimm, Sarah Fahimi, Fabian Kück, Patricia Take, Peer Lauermann, Anna Nguyen-Hoehl, Hans Hoerauf, Nicolas Feltgen, Sebastian Bemme

**Affiliations:** 1 Department of Ophthalmology, University Medical Center Göttingen (UMG Göttingen), Göttingen, Germany; 2 Department of Medical Biometry, University Medical Center Göttingen (UMG Göttingen), Göttingen, Germany

**Keywords:** Macular degeneration, Retinal vein occlusion, Diabetic macular oedema, Optical coherence tomography, Intravitreal injection therapy

## Abstract

**Purpose:**

To investigate the lower visual acuity threshold for recommending intravitreal injection therapy (IVI). The lower limit of 1.3 logMAR best-corrected visual acuity (BCVA) was adopted in 2006 and has been maintained since then.

**Methods:**

In this retrospective study, data from patients with a logMAR BCVA ≤ 1.3 and 24 months follow-up were analysed. We included patients with neovascular age-related macular degeneration (nAMD), diabetic macular oedema (DME), or retinal vein occlusion (RVO).

**Results:**

The data from 164 patients (nAMD: 107; DME: 15; RVO: 42) were analysed. We observed a significant improvement at all time intervals (0 to 6, 6 to 12, 12 to 18, and 18 to 24 months after initiating IVI) compared to baseline. Across all indications, median BCVA improved from 1.4 to 1.0 within the first 6 months and remained stable within 24 months. Patients received a median of 5 and 10 injections within 6 and 24 months, respectively. Median foveal retinal thickness was 594.5 μm at baseline and dropped to 244.5 μm, 235.5 µm, 183 µm, and 180 µm during the four consecutive time intervals.

**Conclusion:**

Patients with nAMD, DME, and RVO with poor baseline BCVA may also benefit from intravitreal therapy with VEGF-inhibitors. In the present study, we observed functional and morphological improvement over 2 years irrespective of the underlying macular disease. Those patients should not be excluded from therapy.

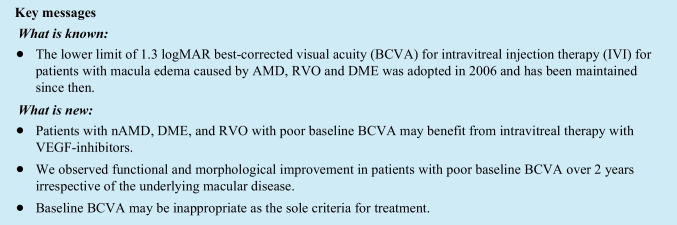

## Introduction

Best-corrected visual acuity (BCVA) is one of the most important parameters in clinical trials for intravitreal injection (IVI) therapy, as functional outcome is the patient’s primary objective. Although there is no threshold at which IVI is no longer considered useful, a BCVA of 1.3 logMAR is recommended from the phase III trials (**nAMD**: (MARINA [6, 7], ANCHOR [37] (Ranibizumab); (VIEW 1&2 [22] (Aflibercept); HAWK & HARRIER  [18–20] (Brolucizumab); TENAYA & LUCERNE [25] (Faricimab), **RVO**: CRUISE [8], BRAVO [13] (Ranibizumab); GALILEO [27, 28, 34], COPERNICUS [5, 9, 23], VIBRANT [14] (Aflibercept), **DME**: RISE & RIDE [9, 33] (Ranibizumab); VIVID & VISTA [11, 24] (Aflibercept), YOSEMITE & RHINE [41] (Faricimab)). This limit has been quite uncritically maintained in clinical routine. Nevertheless, the low vision threshold at which IVI therapy still makes sense remains an unresolved variable. In most of the clinical trials investigating macular diseases, only patients with BCVA > 20/320 (Snellen), equalling a visual acuity of < 1.2 logMAR, were included [6, 7, 18, 19, 22, 38]. On that basis, conclusions are hardly possible about IVI’s effect or benefit in patients with worse baseline visual acuity. Furthermore, it is questionable whether such a limit should be set at all, since IVI therapy’s effects on function and morphology might vary widely amongst individuals [40, 43].

To determine whether patients with low-grade baseline BVCA would benefit from IVI treatment, we retrospectively analysed their functional and morphologic outcome after IVI treatment in patients with a baseline BCVA ≥ logMAR 1.3 with age-related macular disease (AMD), diabetic macular oedema (DME), and retinal vein occlusion (RVO).

## Methods

### Study cohort

In this retrospective study, we enrolled patients undergoing VEGF-inhibitor therapy for nAMD, DME, or RVO, and presenting with a baseline BCVA of ≥ 1.3 logMAR between 2013 and 2020. Only one eye per patient was included; the follow-up period lasted at least 6 months. This study concurs with the 1964 Helsinki declaration and its later amendments, and was approved by our institutional ethics committee (application number is 6/11/20).

All patients with visual acuity ≥ 1.3 logMAR given an injection in the above-mentioned period were included. Patients with better visual acuity were excluded. Both PRN (as needed) and treat-and-extend treatment regimens were included in the study. There were no further exclusion criteria.

### Parameters evaluated

Data was extracted from our institutional database for intravitreal injection therapy. The primary outcome measure was best BCVA during 0 to 6 months, 6 to 12 months, 12 to 18 months, and 18 to 24 months after initiation IVI therapy. BCVA was measured using Snellen charts, and all values were converted in logMAR. Secondary outcome measures included the number of injections and mean central foveal retinal thickness (CFT) at the mentioned time periods. We also recorded the intravitreally administered medication, age, and gender. Furthermore, morphological OCT features were evaluated applying the OCT criteria from the *Orca module* of the Ocean study [26].

### Statistical evaluation

Variables were summarised descriptively for the total cohort and for different groups using absolute and relative frequencies or median with minimum and maximum. For the comparison of the diagnoses, we applied Fisher’s exact test for categorical variables and a rank-based non-parametric test (Brunner et. al 2017) for age and the number of injections. For the analysis of the functional and morphological benefit in different time intervals, we first computed the minimum for BCVA and the mean value for CFT per time interval and patient as representative value. We also computed the mean BCVA per time interval and patient as sensitivity analysis and obtained similar results. Sign tests were performed to compare visual acuity and CFT at these time periods with baseline and to compare the change in BCVA and CFT to zero. We ran McNemar tests to compare IRF (intraretinal fluid) and SRF (subretinal fluid) at different periods to baseline values. Correlation of pre- and best post-therapeutic BCVA was analysed using linear regression. A multilinear regression model was applied to predict the best BCVA and the maximum change in BCVA within the AMD subgroup by BCVA, CFT, and OCT features at baseline. The significance level was set at *α* = 5% for all statistical tests. Due to this study’s exploratory nature, correction for multiple testing was omitted. Analyses were done in the R statistical programming environment (version 3.6.2; R Core Team 2018) using the R package rankFD (version 0.1.1), GraphPad Prism (version 9.1.2), and Microsoft Excel.

## Results

### Study population and treatment

From 3277 patients in our local injection database, we included 164 (5%) patients with BCVA ≥ 1.3 logMAR (Table 1). One hundred seven patients (64.8%) had nAMD, 15 (9.1%) DME, and 42 (26.1%) RVO. The median injection number for all diagnoses was 5 (6 months), 8 (12 months), and 10 (18 and 24 months), with statistically significant differences between diseases for the time interval 6 to 12 months (see Table 1). Most patients were treated with aflibercept (42%), followed by ranibizumab (30%) and bevacizumab (28%) for the first injection. Additional data on IVI-frequency and epidemiology is provided in Table 1.

**Table 1:** Cohort data on patients with VA ≥ 1.3 logMAR

	All diseases	AMD	DME	RVO	*P*
Number of patients (*N*)	164	107	15	42	
Age at first injection Median in years (range)	78 (30–94)	80 (51–94)	67 (30–84)	76 (33–93)	** < 0.0001**
Gender: female/male *N* (%)	87/77 (53.0/47.0)	57/50 (53.3/46.7)	7/8 (46.7/53.3)	23/19 (54.8/45.2)	0.913
Eye: right/left (%)	77/87 (47.0/53.0)	49/58 (45.8/54.2)	8/7 (53.3/46.7)	20/22 (52.4/47.6)	0.886
Number of injections within time periods after initial IVI Median (range)					
0 to 6 months	5 (2–7)	5 (3–7)	6 (2–7)	5 (3–7)	0.341
6 to 12 months	3 (0–7)	3 (0–6)	2 (0–4)	3 (1–7)	**0.006**
12 to 18 months	2 (0–6)	2 (0–6)	0 (0–4)	2 (0–5)	0.075
18 to 24 months	0 (0–5)	0 (0–5)	0 (0–5)	0 (0–5)	0.614

The values in bold are statistically significant. There was a significant age difference between patients with AMD, DME and RVO. In addition, there was a significant difference in the number of injections between patients with DME, AMD, and RVO in the 2nd half of the year

### Visual outcome

**Fig. 1:**
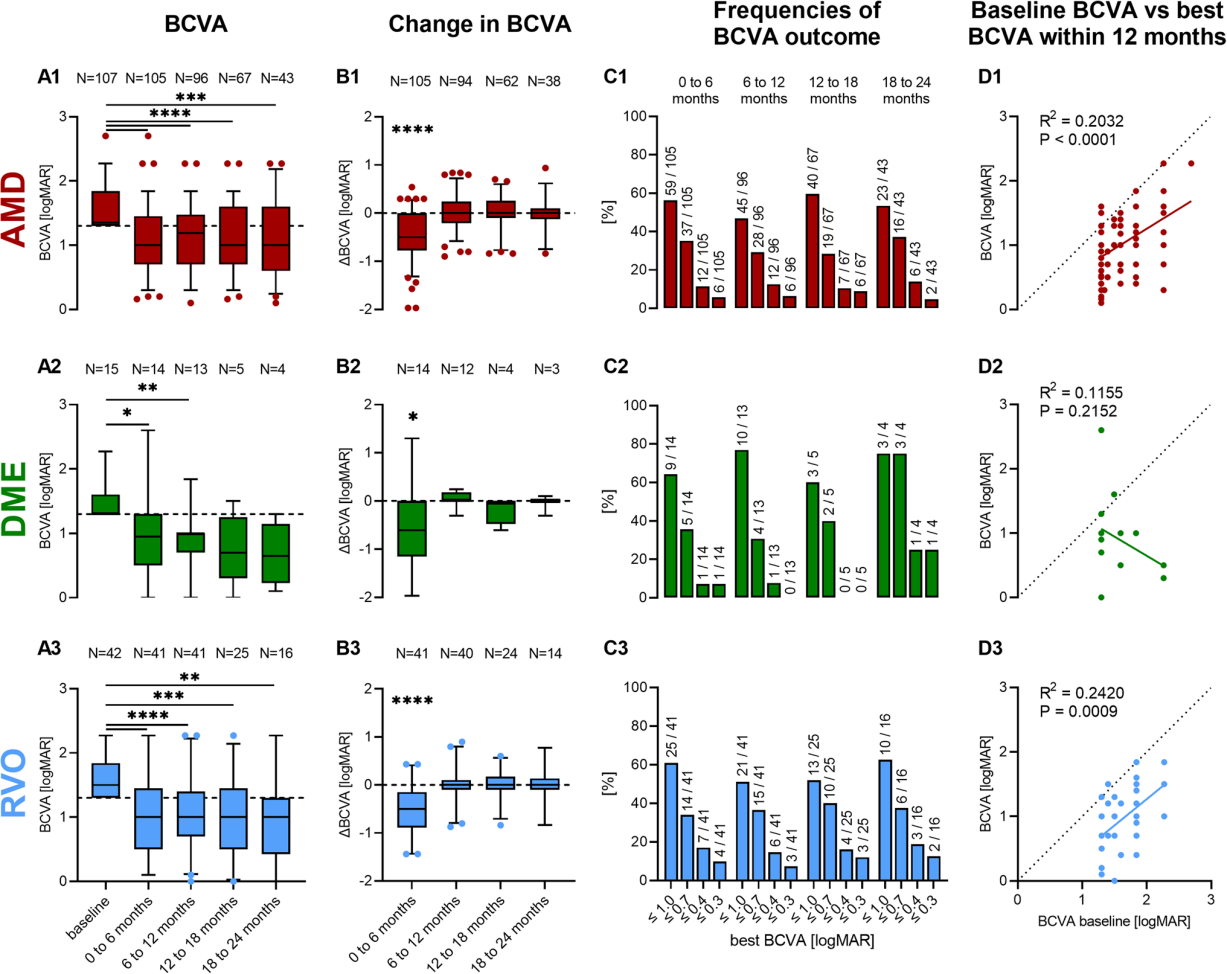
BCVA (**A1**–**A3**) and change in BCVA (**B1**–**B3**) of AMD (red), DME (green) and RVO (blue) patients with baseline BCVA of ≥ 1.3 logMAR (dashed line). Best BCVA at various time intervals (0 to 6, 6 to12, 12 to 18, and 18 to 24 months) after starting intravitreal anti-VEGF therapy was compared to baseline (sign test), and maximum change in BCVA was compared to zero (sign test). (**C1**–**C3**) Relative frequencies (bars) and actual counts (annotations above bars) of patients whose BCVA reached a defined threshold of ≤ 1.0, ≤ 0.7, ≤ 0.4 or ≤ 0.3 logMAR at the different time intervals. (**D1**–**D3**) Correlation between initial visual acuity and the best BCVA within 12 months. *P < 0.05, **P < 0.01, ***P < 0.001, ****P < 0.0001

The median BCVA (logMAR) was 1.4 at initial presentation and improved to 1.0 within the first 6 months. During the subsequent periods, median BCVA remained stable measuring 1.0 at all intervals after first IVI. Baseline BCVA (median, logMAR) was similar amongst the three groups with 1.35 (range 1.3–2.7) in AMD patients, 1.3 (range 1.3–2.3) in DME patients, and 1.5 (range 1.3–2.3) in RVO patients. Best BCVA improved significantly to 1.0 (range 0.2–2.7) in AMD (*P* < 0.0001), 0.95 (range 0.0–2.6) in DME (*P* = 0.019), and 1.0 (range 0.1–2.3) in RVO (*P* < 0.0001) within the first 6 months after initiating VEGF-inhibitor therapy. Boxplots in Fig. 1 (A1–A3) illustrate the BCVA distribution in each group at baseline and during the various follow-up periods. Maximum change in BCVA (median, logMAR) during the first 6 months measured − 0.5 in AMD patients, − 0.6 in DME, and − 0.5 in RVO patients and differed significantly from zero within all groups (Fig. 1, B1–B3). During the following periods, we did not detect any significant BCVA changes in any of the three groups. Subgroup analysis revealed that patients with branch retinal vein occlusion (BRVO) benefited most, showing a BCVA improvement by − 0.64 (median, logMAR) during the first 6 months after first IVI.

To enable a more detailed view on the distribution of BCVA outcomes, the percentages of patients with a BCVA of ≤ 1.0, ≤ 0.7, ≤ 0.4, and ≤ 0.3 are displayed in Fig. 1 (C1–C3) for each period. During the first 6 months, over 30% of patients achieved a visual acuity of ≤ 0.7 logMAR regardless of their diagnosis. Approximately 5% of all AMD and DME patients and 10% of all RVO patients managed to achieve a visual acuity of 0.3 logMAR or better. Counts of analysed BCVA values displayed in Fig. 1 (A1–A3) constantly decreased from *N* = 107 (AMD), 15 (DME), and 42 (RVO) at baseline to *N* = 43 (AMD), 4 (DME), and 16 (RVO). However, the distribution of BCVA outcomes (Fig. 1, C1–C3) remained similar across all periods.

To test whether pre-therapeutic visual function alone is sufficient to predict visual outcome following VEGF-inhibitor therapy in our study, we correlated baseline BCVA and best BCVA within 12 months (Fig. 1, D1–D3). We noted an only moderately strong association between pre- and best post-therapeutic BCVA in AMD and RVO patients, and no significance in DME patients, data that is also reflected by the wide range of BCVA change from pre- to best post-therapeutic values within 12 months (AMD: − 1.97 to 0.30; DME: − 1.97 to 1.30; RVO − 1.50 to 0.10).

### OCT morphology

OCTs were evaluated using the *Orca module*, which assesses certain morphological OCT parameters listed in Table 2 [26]. Seventy-seven of the 164 eyes with BCVA of ≥ 1.3 logMAR were included in the OCT evaluation. In the remaining cases, we either had no access to the OCT data, or evaluation was limited due to fixation artefacts or poor image quality. In the past, when treatment decisions were being made, most of the OCT data was available and was used to inform treatment. In the few cases in the past where the OCT data could not be fully evaluated, we made every effort to consider other clinical factors such as visual acuity, symptom severity, and fundoscopy findings in order to inform treatment decisions. Of the 77 eyes included, 83.1% revealed intraretinal fluid (IRF), 70.1% subretinal fluid (SRF), 49.4% pigment epithelial detachment, 46.4% posterior vitreous detachment, 36.4% epiretinal gliosis, 15.6% subretinal fibrosis, 15.6% pigment epithelial atrophy, 2.6% vitreomacular traction, 2.6% vitreous haemorrhage, and 1.3% pigment epithelial rupture at baseline. Additional subgroup analyses of OCT characteristics for the three different macular diseases separately are shown in Table 2.

**Table 2:** Morphology of baseline OCT parameters using the *Orca module*

Parameter	All diseases	nAMD	DME	RVO
*N*	77	47	4	26
Vitreous haemorrhage	2 (2.6%)	2 (4.3%)	0	0
Vitreous detachment	32 (46.4%) ^*#*^*Missing 8*	31 (72.1%) ^*#*^*Missing 4*	1 (50%) ^*#*^*Missing 2*	0 ^*#*^*Missing 2*
Vitreomacular traction	2 (2.6%)	1 (2.1%)	1 (25%)	0
Epiretinal gliosis	28 (36.4%)	18 (38.3%)	3 (75%)	7 (26.9%)
Intraretinal fluid	64 (83.1%)	35 (74.5%)	3 (75%)	26 (100%)
Subretinal fluid	54 (70.1%)	39 (83%)	0	15 (57.7%)
Pigment epithelial detachment (PED)	38 (49.4%)	36 (76.6%)	1 (25%)	1 (3.8%)
Pigment epithelium tears (PE tear)	1 (1.3%)	1 (2.1%)	0	0
Subretinal fibrosis	12 (15.6%)	11 (23.4%)	0	1 (3.8%)
Geographic atrophy (GA)	12 (15.6%)	11 (23.4%)	1 (25%)	0

^#^In the missing cases, a reliable evaluation of the vitreoretinal interface was impossible

**Fig. 2:**
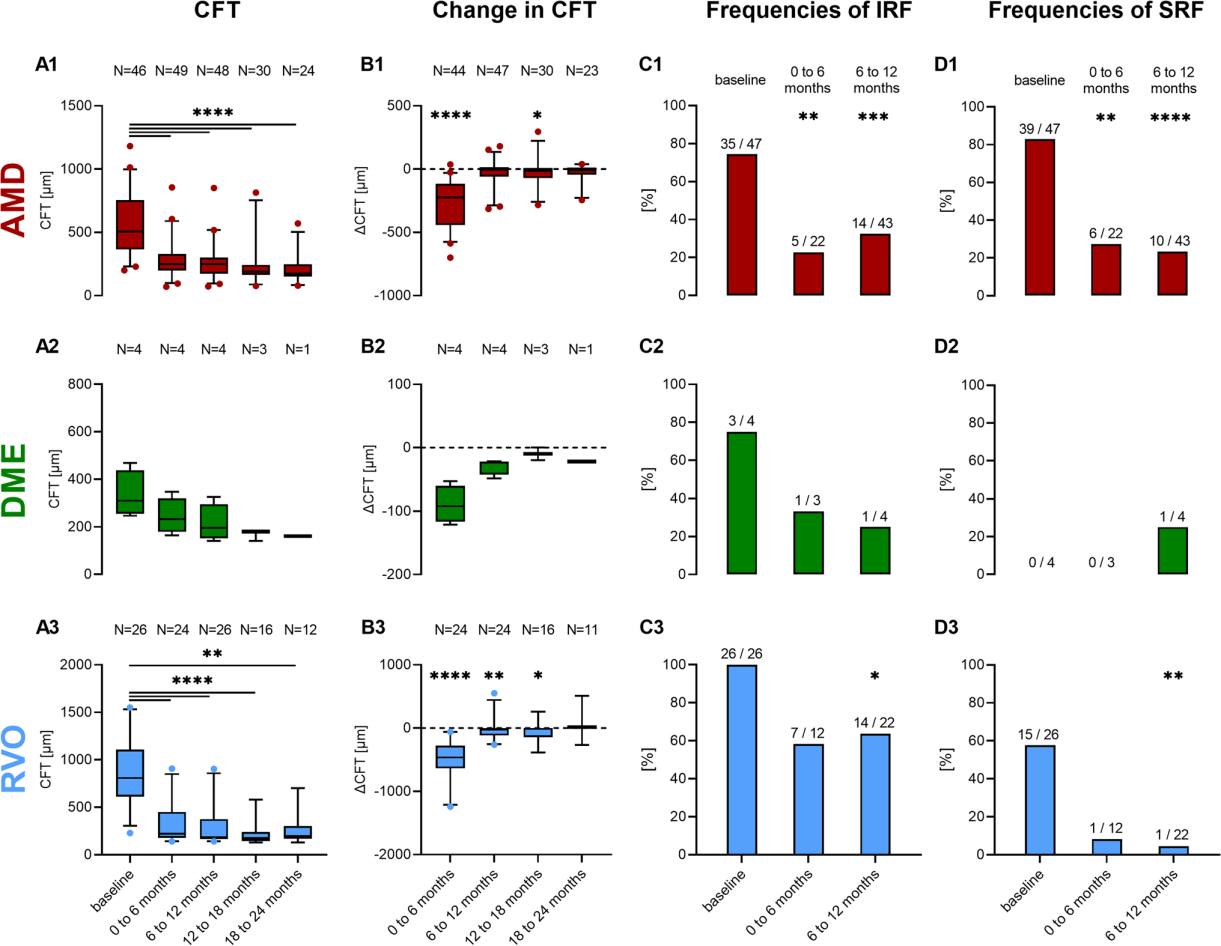
CFT (**A1**–**A3**) and change in CFT (**B1**–**B3**) of AMD (red), DME (green), and RVO (blue) patients. CFT at various time intervals (0 to 6, 6 to 12, 12 to 18, and 18 to 24 months) after starting intravitreal VEGF-inhibitor therapy was compared to baseline (sign test), and change in CFT was compared to zero (sign test). Relative frequencies (bar) and actual counts (annotation above bars) of patients with IRF (**C1**–**C3**) and SRF (**D1**–**D3**) on SD-OCT. IRF and CRF frequencies during the 0- to 6-month and 6- to 12-month period were compared to baseline (McNemar test). *P < 0.05, **P < 0.01, ***P < 0.001, ****P < 0.0001

Amongst all diagnoses, median central foveal thickness (CFT) decreased from 594.5 μm at baseline to 244.5 μm from 0 to 6 months, to 235.5 μm from 6 to 12 months, to 183 μm from 12 to 18 months, and to 180 μm from 18 to 24 months. Boxplots in Fig. 2 illustrate the CFT distribution (A1–A3) as well as change in CFT (B1–B3) in each group at baseline and during the various follow-up periods. Of the parameters in Table 2, IRF and SRF were analysed in more detail (see Fig. 2C and D). At initial presentation, 83% exhibited SRF and 70% IRF of the 77 analysed OCTs. We detected both IRF and SRF in significantly fewer patients during the 0- to 6-month (IRF: 35.1%, SRF 18.9%, *P* < 0.001) and during the 6- to 12-month period (IRF: 42.0%, SRF 17.4%, *P* < 0.0001).

### Predicting BCVA based on baseline functional and morphological parameters for AMD patients

Since our data revealed only a moderately strong association between pre- and post-therapeutic BCVA on univariate analysis, we further tested if predicting functional outcome in our population might improve by including biomarkers of baseline SD-OCT morphology. To do so, we performed a multilinear regression on the AMD subgroup to predict the best BCVA as well as the maximum change in BCVA including baseline BCVA, baseline CFT, and the presence of IRF, SRF, PED, pigment epithelium tears, subretinal fibrosis, or geographic atrophy (GA) on baseline SD-OCT. The association between baseline BCVA and the best post-therapeutic BCVA within 12 months remained significant. Additionally, SRF and subretinal fibrosis revealed a significant impact on best BCVA within 12 months and thus also on the maximum change in BCVA. Furthermore, there was a trend towards a larger improvement in BCVA for patients with worse initial BCVA (*β* =  − 0.33, 95% CI: − 0.72 to − 0.06, *P* = 0.093). In summary, our multiple linear regression analysis of the AMD subgroup revealed that patients with better initial BCVA, without SRF, and without subretinal fibrosis on baseline SD-OCT achieved a better BCVA outcome. The results of the multilinear regression model on the best BCVA are summarised in Table 3.

**Table 3:** Multilinear regression model on BCVA within the AMD subgroup (number of observations: 45)

Parameter at baseline	BCVA		
	*β*	95% CI	*P*
Intercept	− 0.6610	− 1.590 to 0.2679	0.1576
BCVA	0.6693	0.2813 to 1.0570	**0.0013**
CFT	0.0004	− 0.0002 to 0.0010	0.1916
IRF	0.0253	− 0.2665 to 0.3171	0.8614
SRF	0.4553	0.1235 to 0.7870	**0.0085**
PED	− 0.0300	− 0.3834 to 0.3235	0.8644
PE tear	0.0097	− 0.8617 to 0.8810	0.9822
Fibrosis	0.3556	0.0170 to 0.6942	**0.0401**
GA	0.0254	− 0.3010 to 0.3518	0.8756

There is a statistically significant correlation for the values printed in bold. Patients with better initial BCVA, without SRF and without subretinal fibrosis achieved a better BCVA outcome

## Discussion

In this retrospective study, we analysed the functional and morphological benefit in patients with low baseline BCVA of ≥ 1.3 logMAR with AMD, DME or RVO. Across all the diagnoses, BCVA improved significantly to 1.0 logMAR after initiating IVI therapy and remained stable up to 24 months of follow-up. RVO patients presenting poor baseline visual acuity actually benefitted more than our AMD and DME patients. Five percent of all AMD and DME patients and 10% of all RVO patients achieved a visual acuity of 0.3 logMAR or better, which corresponds to reading visual acuity. Our data highlight the need to treat patients presenting a low baseline visual acuity of ≥ 1.3 logMAR, as they not only have a realistic chance of regaining functional visual acuity—such an improvement means they can achieve visual acuity far beyond orientation vision. Considering that over a million IVI treatments are applied per year in Germany, a considerable number of affected patients might be deprived of therapy despite being able to benefit significantly from therapy [42].

Our results reveal the question as to whether it would be worthwhile to re-assess the lower limit for IVI therapy. Our study cohort’s improvement in BCVA demonstrated high inter-individual variability and only a moderately strong association between pre- and post-therapeutic BCVA, thus arguing against a lower limit for IVI therapy at all. A multiple linear regression analysis of our AMD subgroup showed that baseline BCVA had a significant impact on BCVA outcome, but not on the extent of change in BCVA, i.e. an AMD patient with a low baseline BCVA of 1.8 logMAR might have a similar (or even larger) BCVA improvement as one with a baseline BCVA of 1.3 logMAR. Our data provides evidence supporting that the treatment decision should not only depend on baseline BCVA as the sole criterion in low-vision patients, especially in those with AMD or RVO. In the DME subgroup, our assessment was limited due to small group size.

Basing the decision for IVI treatment in low-vision patients on function by relying on baseline BCVA might entail a significant risk of misjudgment. In clinical practice, visual acuity is often tested and documented in individuals with severe visual impairment by asking the patient to count fingers. To improve the decision making for IVI therapy in a clinical setting based on BCVA in low-vision patients, it might be more appropriate to apply more accurate visual acuity testing, e.g. via EDTRS charts or the Freiburg Visual Acuity Test (FrACT), in [30, 39]. Another aspect that argues against a generally applied lower limit for IVI therapy is a potentially better response with newer substances, a factor that needs to be tested separately in patients with low baseline BCVA [18, 19, 25]. Moreover, there are special indications such as central subretinal haemorrhage complications in the nAMD setting where initial visual acuity is usually below the 1.3 logMAR threshold (Bopp et al. 2012).

Regarding morphological benefit, our data showed a significant reduction in retinal thickness accompanied by significant reduction in the frequency of intraretinal and subretinal fluid, thus indicating a good morphological response to IVI therapy in our low-vision patients. However, multiple linear regression analysis showed that baseline CFT and the presence of IRF on baseline SD-OCT had no significant impact on BCVA outcome in the AMD subpopulation. The presence and extent of intraretinal oedema might be less helpful when making therapy decisions for low-vision AMD patients. In general, AMD patients with SRF on SD-OCT reveal a more benign course than those with IRF [36]. Interestingly, in our study, patients with SRF present on baseline SD-OCT achieved a worse BCVA outcome, making the role of SRF on baseline OCT in low-vision AMD controversial. Less surprisingly, subretinal fibrosis showed a significant impact on BCVA outcome in our AMD subgroup. This data underscores how necessary it is to include baseline OCT pathologic features within the decision process for IVI therapy in low-vision patients.

Our study has some limitations. The evaluation of IVI was carried out retrospectively. In particular, the dropout rate after more than 12 months after initiation IVI therapy was high. Since BCVA and CFT might become worse after dropout, we thus cannot draw clear, reliable conclusions for later time points. However, we would expect the improvement seen during the first 12 months to persist after 24 months.

Only 55 patients completed the study at 24 months, as noted in the figures and tables. This number of patients who completed the study at 24 months may have been influenced by a variety of factors, including treatment adherence, adverse events, and patient preferences.

Another limitation is the relatively small sample size for the multivariable regression analysis. The results should be interpreted accordingly. In conclusion, patients suffering from severe visual impairment caused by AMD, RVO, and probably DME might benefit significantly and should thus be considered for IVI therapy when no obviously irreversible damage like extended subretinal fibrosis is present. Otherwise, neglecting this patient group unjustly would deprive them of a chance for visual improvement. Furthermore, we maintain that baseline BCVA is inappropriate as the sole criterion to treat or not to. More accurate visual acuity testing and progress in evaluating OCT biomarkers, as well as including patients with BCVA ≥ 1.3 logMAR in future studies are essential to improve this decision-making process. Ophthalmologists will eventually have to discuss the benefit-risk profile, expectations, and visual prognosis with each patient individually. The treatment decision should be reviewed and constantly re-evaluated during IVI-therapy.
